# A genome-wide CRISPR/Cas9 screen to identify phagocytosis modulators in monocytic THP-1 cells

**DOI:** 10.1038/s41598-021-92332-7

**Published:** 2021-06-21

**Authors:** Benjamin Lindner, Eva Martin, Monika Steininger, Aleksandra Bundalo, Martin Lenter, Johannes Zuber, Michael Schuler

**Affiliations:** 1 Department of Drug Discovery Science, Boehringer Ingelheim Pharma GmbH & Co. KG, Birkendorferstr. 65, 88397 Biberach an der Riss, Germany; 2 Research Institute of Molecular Pathology (IMP), Vienna BioCenter (VBC), 1030 Vienna, Austria; 3 Medical University of Vienna, Vienna BioCenter (VBC), 1030 Vienna, Austria

**Keywords:** Innate immune cells, Innate immunity, CRISPR-Cas9 genome editing, High-throughput screening, Bacterial host response

## Abstract

Phagocytosis of microbial pathogens, dying or dead cells, and cell debris is essential to maintain tissue homeostasis. Impairment of these processes is associated with autoimmunity, developmental defects and toxic protein accumulation. However, the underlying molecular mechanisms of phagocytosis remain incompletely understood. Here, we performed a genome-wide CRISPR knockout screen to systematically identify regulators involved in phagocytosis of *Staphylococcus* (*S*.) *aureus* by human monocytic THP-1 cells. The screen identified 75 hits including known regulators of phagocytosis, e.g. members of the actin cytoskeleton regulation Arp2/3 and WAVE complexes, as well as genes previously not associated with phagocytosis. These novel genes are involved in translational control (EIF5A and DHPS) and the UDP glycosylation pathway (SLC35A2, SLC35A3, UGCG and UXS1) and were further validated by single gene knockout experiments. Whereas the knockout of EIF5A and DHPS impaired phagocytosis, knocking out SLC35A2, SLC35A3, UGCG and UXS1 resulted in increased phagocytosis. In addition to *S. aureus* phagocytosis, the above described genes also modulate phagocytosis of *Escherichia coli* and yeast-derived zymosan A. In summary, we identified both known and unknown genetic regulators of phagocytosis, the latter providing a valuable resource for future studies dissecting the underlying molecular and cellular mechanisms and their role in human disease.

## Introduction

Phagocytosis is a specialized form of endocytosis by which a cell engulfs solid particles (> 0.5 µm) from the extracellular space within a plasma membrane envelope^1^. Microorganisms, dead or dying cells, and cell debris can be taken up by phagocytosis. Once internalized, the engulfed particles are contained within a phagosome which then fuses with lysosomes for degradation. Phagocytosis of microbial pathogens, apoptotic and necrotic cells contributes to tissue homeostasis by mediating pathogen elimination and clearance of damaged cells. Classical phagocytosing cells are monocytes, macrophages, neutrophils, dendritic cells, osteoclasts and eosinophils^2^. Additionally, fibroblasts, as well as epithelial and endothelial cells, can perform phagocytosis of apoptotic cells^3^. The importance of phagocytosis in both health and disease becomes evident in cases of imbalanced phagocytosis, which harbors the potential to lead to autoimmunity, developmental defects or accumulation of toxic protein aggregates^3–5^.The mechanisms of particle internalization depend on size and type of the particle, as well as the location of the phagocyte^6^. To ensure properly conducted phagocytosis, it is essential that phagocytes differentiate between living and senescent cells, infected and uninfected cells, pathogens and commensals as well as different classes of foreign particles, e.g. gram-positive and -negative bacteria^1^. Foreign particles, like microbial pathogens displaying surface molecules not found in higher organisms can directly interact with phagocyte receptors. For the phagocytosis of bacteria, various microbial proteins, glycoconjugates, lipopolysaccharides, lipoteichoic acids and mycobacterial lipids are essentially required^1^. For example, the C-type lectin receptor CLECSF8 is a key component of anti-microbial host defense, and mice lacking this receptor show an increased bacterial burden^7^. In contrast, phagocytic removal of e.g. apoptotic cells turned out to be more complex, with exposure of a plethora of ‘eat me’ signals on the surface of the dying cell triggering phagocytosis. For efficient recognition of target particles, receptors on the phagocyte interact with ligands present on the particle, enabling engulfment and finally its uptake^2,3,6^. Subsequently, phagocytosis initiates a series of intracellular fusion and fission events, which ultimately result in the formation of the phagolysosome^8^. Acidification by vacuolar ATPases leads to a decrease in the phagolysosomal pH, which further facilitates degradation of the ingested particle^2,9,10^.

The high level of redundancy of both phagocytosis ligands and receptors reflects the importance and complexity of the process and complicates the genetic elucidation of involved components. Classically, phagocytosis has been studied using cell biology and microscopy techniques, partially combined with genetic manipulation of individual genes. Systematic analysis of the genes required for phagocytosis using genetic screening technologies has been described for *C. elegans* and *D. melanogaster,* and several genes with mammalian orthologues performing analogous functions could be identified^11–15^. However, until recently^16,17^, systematic screening of mechanisms regulating mammalian phagocytosis has not been reported.

In the last years, pooled genetic screens using either RNA interference or CRISPR Cas9-mediated gene knockouts became popular tools to investigate thousands of perturbations in a single experiment^18^. Pooled CRISPR screens allow for effective and systematic interrogation of complex cellular processes when combined with appropriate selection strategies. Here, we describe our efforts to identify genes regulating phagocytosis of bacteria in THP-1 cells, a human leukemia cell line with morphological and functional properties of primary monocytes^19^. We used our previously established FACS-based phagocytosis assay^20^ and performed a genome-wide CRISPR screen in THP-1 cells phagocytosing *S. aureus particles.* Subsequently, the selected hits were validated in single gene knockout experiments and characterized with additional phagocytosis target particles.

## Results

To identify genes regulating phagocytosis of the bacterium *S. aureus*, we selected the human monocytic cell line THP-1^19^ as our primary screening model. THP-1 cells have been shown to spontaneously phagocytose bacteria without the need for activation or differentiation^20^. To establish THP-1 cells as a screening model which ensures homogenous Cas9 expression and thereby a high dynamic range of effect sizes in CRISPR screens^21^, we engineered them to harbor a tetracycline (Tet)-inducible Cas9-GFP expression system, isolated single cell-derived clones, and characterized them for inducible Cas9 expression (iCas9), homogenous CRISPR/Cas9 editing, and their phenotypic properties. Among several clones showing homogenous Cas9-GFP induction (Supplementary Figure S1a) and efficient knockout of the surface marker CD46 upon expression of a CD46-specific sgRNA (Supplementary Figure S1b), we selected five clones for further characterization. Compared to unperturbed THP-1 bulk cells, all five THP-1 iCas9 clones grew at enhanced rates (Supplementary Figure S1c) while displaying similar or even enhanced expression of the monocyte-associated surface markers CD11b, CD36, and CD14 (Supplementary Figure S1d). From these clones, we selected one clone (A2) as our screening model and further tested its functionality using the CRISPR library vector (sgETN), which co-expresses murine Thy1.1, a surface protein that can be used for magnetic-activated cell sorting (MACS) enrichment of transduced cells. To further evaluate the efficacy of CRISPR mutagenesis, we transduced THP-1 iCas9 (clone A2) with a pool of sgETN-sgRNAs targeting 5 essential genes (TIMELESS, WDHD1, RAD21, SMC3, PLK1) or non-targeting controls, partially enriched transduced cells using MACS (to ~ 60% Thy1.1^+^), induced Cas9 expression using doxycycline (dox) treatment, and monitored the fraction of Thy1.1^+^/sgRNA-expressing cells over time. While the fraction of Thy1.1^+^ non-targeting control sgRNA-expressing cells remained stable over time, Cas9 induction led to a strong depletion of cells transduced with sgRNAs targeting essential genes (reaching 36-fold on day 13), indicating effective CRISPR mutagenesis in the vast majority of cells (Supplementary Figure S1e). In a last step, we tested whether an optimized phagocytosis assay previously established by our group^20^ detects known phagocytosis regulators in the selected THP-1 iCas9 clone. To this end, THP-1 iCas9 cells were transduced with a non-targeting control sgRNA or an sgRNA targeting ARPC4, whose loss of function has recently been reported to impair phagocytosis in U937 cells^16^. Indeed, CRISPR-mutagenesis of ARPC4 reduced the fraction of phagocytosing cells by more than fourfold compared to the non-targeting control sgRNA (Supplementary Figure S2), demonstrating that our assay recapitulates the function of established phagocytosis regulators.

**Figure 1:**
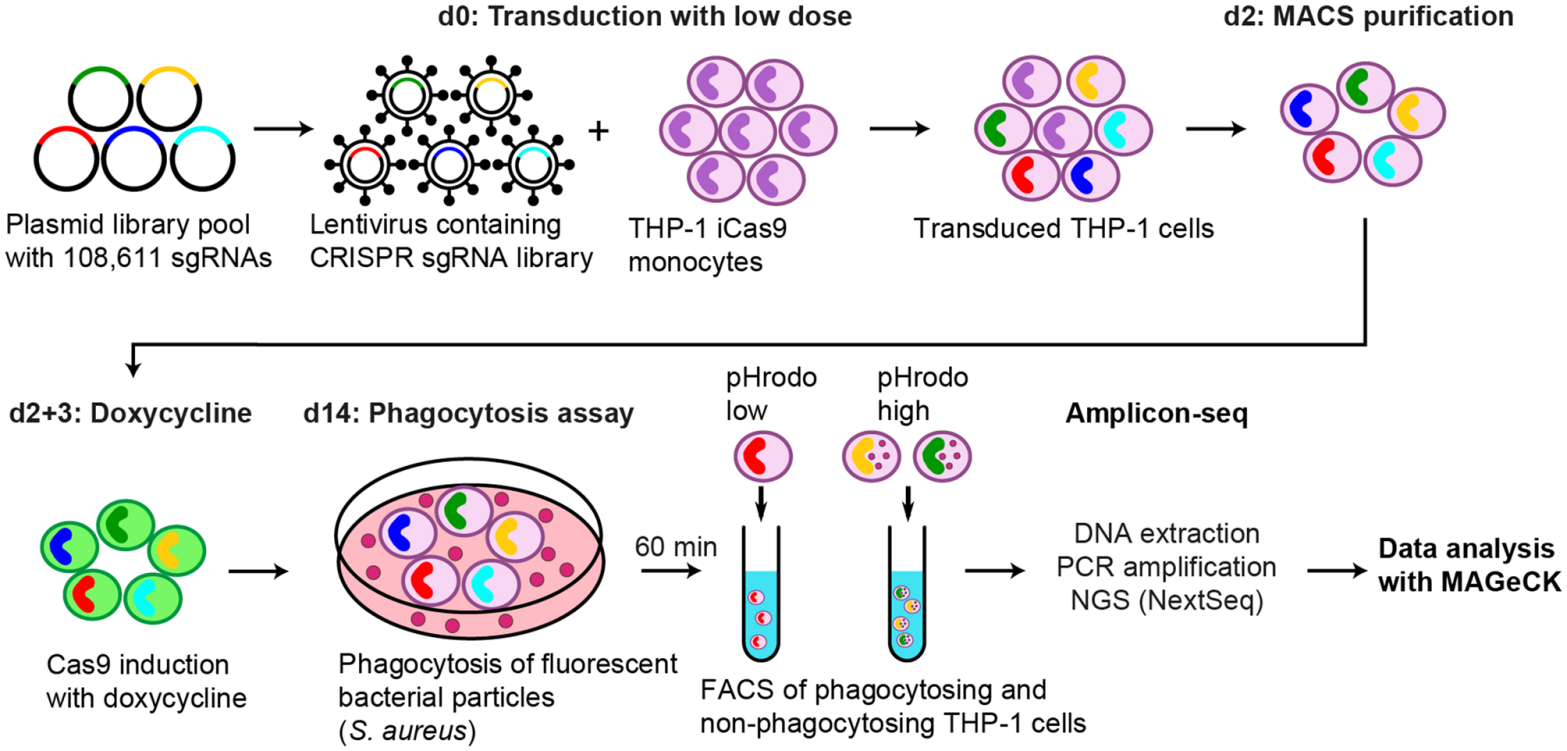
Genome-wide CRISPR screen for genetic regulators of phagocytosis of bacteria. THP-1 iCas9 cells were transduced at a coverage of 2919× with a genome-wide pooled sgRNA library containing 6 sgRNAs per gene and 500 non-targeting controls. Transduced cells were enriched by MACS and Cas9 was induced with dox on two consecutive days. 14 days after transduction, *S. aureus* particles labeled with pHrodo red was added to the cells. After 60 min, cells were sorted by FACS according to their fluorescence intensity in phagocytic active and inactive populations. The integrated sgRNAs were amplified and sequenced with NextSeq 550. Sequencing data was analyzed with MAGeCK-VISPR.

Having established a suitable cellular model and FACS-based assay for CRISPR/Cas9-based identification of phagocytosis regulators, we performed a genome-wide CRISPR screen following the workflow depicted in Fig. 1. THP-1 iCas9 cells were transduced with a genome-wide sgRNA library containing 6 guides per gene and 500 non-targeting controls (18,187 genes, ~ 6 sgRNA per gene and 108,611 sgRNAs total^21^) with a transduction rate of 33.9% and a cell to guide coverage of approximately 3000×. The screen was performed in duplicates and triplicates for the baseline and phagocytosis samples, respectively. For each replicate a library coverage of more than 1000× was maintained throughout the experiment to mitigate a major source of experimental noise^18^. After MACS enrichment of transduced cells, baseline samples were collected before induction of Cas9 expression. Genome-wide knockout effects on phagocytosis were assessed 12 days post Cas9 induction by addition of commercially available pHrodo red labeled *S. aureus* particles. After 1 h of incubation, cells were collected and sorted according to their pHrodo signal intensity. The purity of the sorted cell populations was about 98% (Supplementary Figure S3). The DNA of the sorted cell populations was isolated, integrated sgRNAs were amplified by PCR and sequenced using Illumina’s NextSeq platform. The sgRNA counts of the raw reads from each sample were determined (Supplementary Table S3) and further analyzed using MAGeCK-VISPR^22,23^ (Supplementary Table S4). Secondary analysis including filtering, comparisons, and plotting was performed in RStudio^24^. As a quality control, we also sequenced the lentiviral vector plasmid pool. About 99% of the designed sgRNAs were detected in the library and the reads of cloned sgRNAs were evenly distributed with only few high copy outliers (Supplementary Figure S4a). The relative difference in the sgRNA abundance in the library was very low and 80% of the sgRNAs were present with counts of less than tenfold difference (Supplementary Figure S4b).

Amplicon-seq of populations collected throughout the screen revealed high mapping rates (no mismatches allowed) of > 80% (Supplementary Figure S5a). As expected, the number of missing sgRNAs and the gini index of the phagocytosing and non-phagocytosing samples were higher compared to the baseline samples (Supplementary Figure S5b and c). The gini index is a measure of unevenness, smaller numbers indicate more evenness and accordingly higher numbers indicate more unevenness. The correlation between phagocytosing and non-phagocytosing samples is rather high (Pearson: 0.93–0.97), but clearly separates from the baseline samples (Supplementary Figure S5d). As the effects of gene editing on the rate of phagocytosis were assessed 12 d post Cas9 induction, we assumed that sgRNAs targeting essential genes (constitutive core essential genes as defined in Hart et al.^25^) might be depleted. Therefore, sgRNAs were grouped into non-targeting controls (NTC), targeting essential genes (211), and targeting the remaining genes (17,976), and the distribution of these groups throughout the screen was analyzed. In the plasmid pool and baseline samples all three groups are equally distributed, demonstrating a uniform transduction of the sgRNA library. In contrast, the FACS enriched populations (the normalized data of all phagocytosing and non-phagocytosing samples combined) have decreased sgRNA counts targeting essential genes compared to the non-targeting and remaining, non-essential sgRNAs (Supplementary Figure S5e).

**Figure 2:**
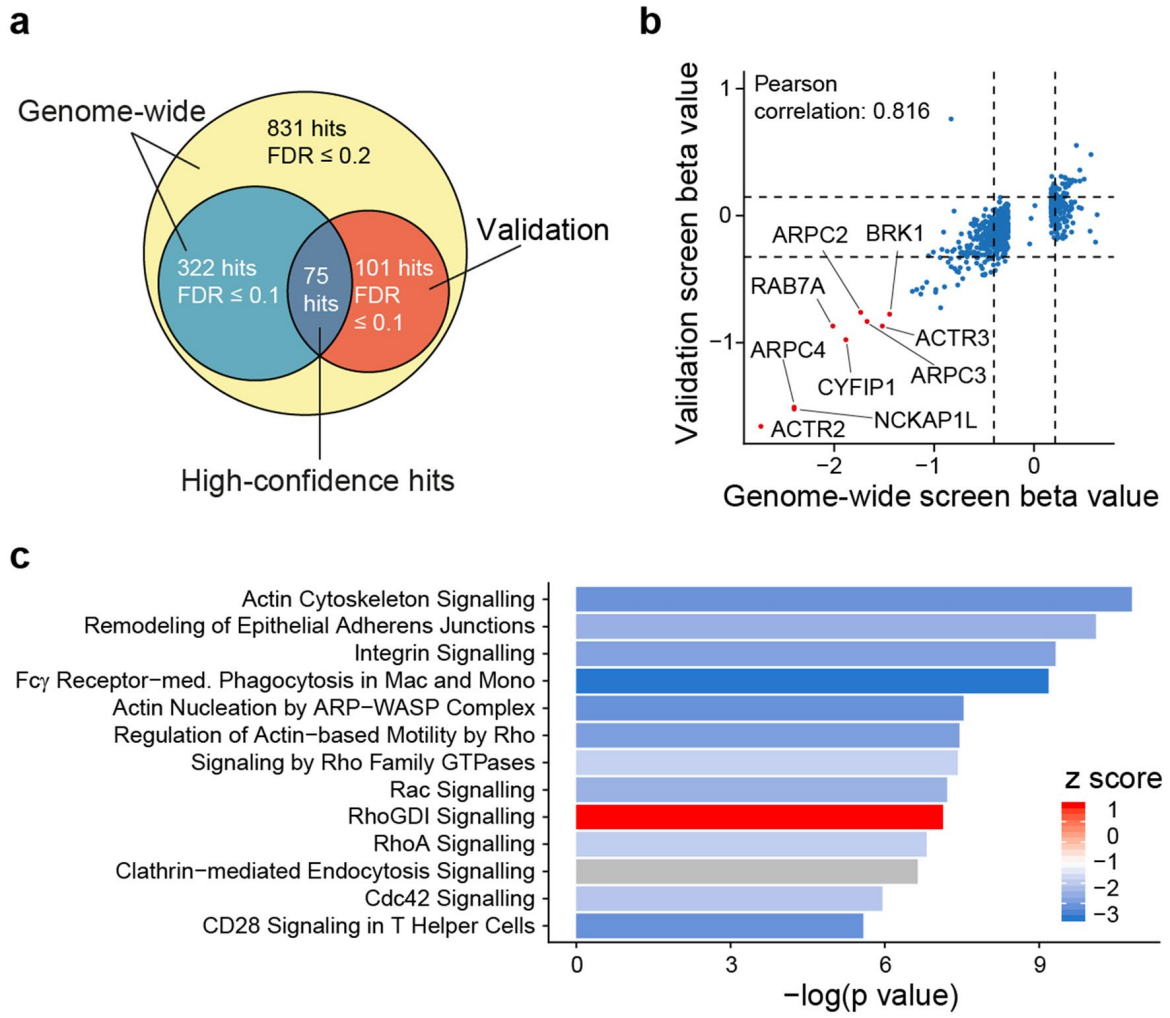
Comparison of both screens and secondary analysis of high confidence hits.** (a)** A second screen targeting a subset of genes (831 genes with FDR ≤ 0.2) was performed at very high cell-to-guide coverage (36,000×). 75 genes were detected with FDR ≤ 0.1 in both screens. One gene from the first screen showed an opposite effect in the 2nd screen and was excluded. **(b)** Beta values of the second screen are compared to the beta values of the first screen. **(**c**)** Depicted are enriched canonical pathways from IPA with − log(p values) of ≥ 5. The z score is color coded; positive and negative values indicate inhibition and activation of phagocytosis, respectively.

Comparing the sgRNA counts of sorted phagocytosing to non-phagocytosing cell populations using MAGeCK with an FDR of ≤ 0.2 resulted in a list of 831 genes, which was used to generate an sgRNA pool for a second screen, aiming to increase the confidence of the primary hits (Fig. 2a). In the second screen, a much higher cell to guide coverage was used to reduce the risk of sgRNA dropouts by chance. THP-1 iCas9 cells were transduced with a lentiviral vector pool of 5407 sgRNAs (~ 6 guides per gene and 500 non-targeting controls) at a coverage of > 36,000 cells per guide, which was kept throughout the screen. Again, transduced cells were enriched, Cas9 expression was induced and phagocytosing and non-phagocytosing cells were sorted as described before. The quality of the screen metrics (mapping rate, missed sgRNAs) was equal or even better than in the first screen with an improved separation of non-/phagocytosing samples from the baseline samples (Supplementary Figure S6a–d, Supplementary Table S5). Still, phagocytosing and non-phagocytosing samples were highly correlating (Supplementary Figure S6d). The decrease in sgRNA counts targeting essential genes was comparable with the first screen (Supplementary Figure S6e). The distribution of sgRNAs in the validation pool was very even with no missing sgRNA and 80% of sgRNAs present with counts of less than sixfold difference (Supplementary Figure S4c, d).

**Figure 3:**
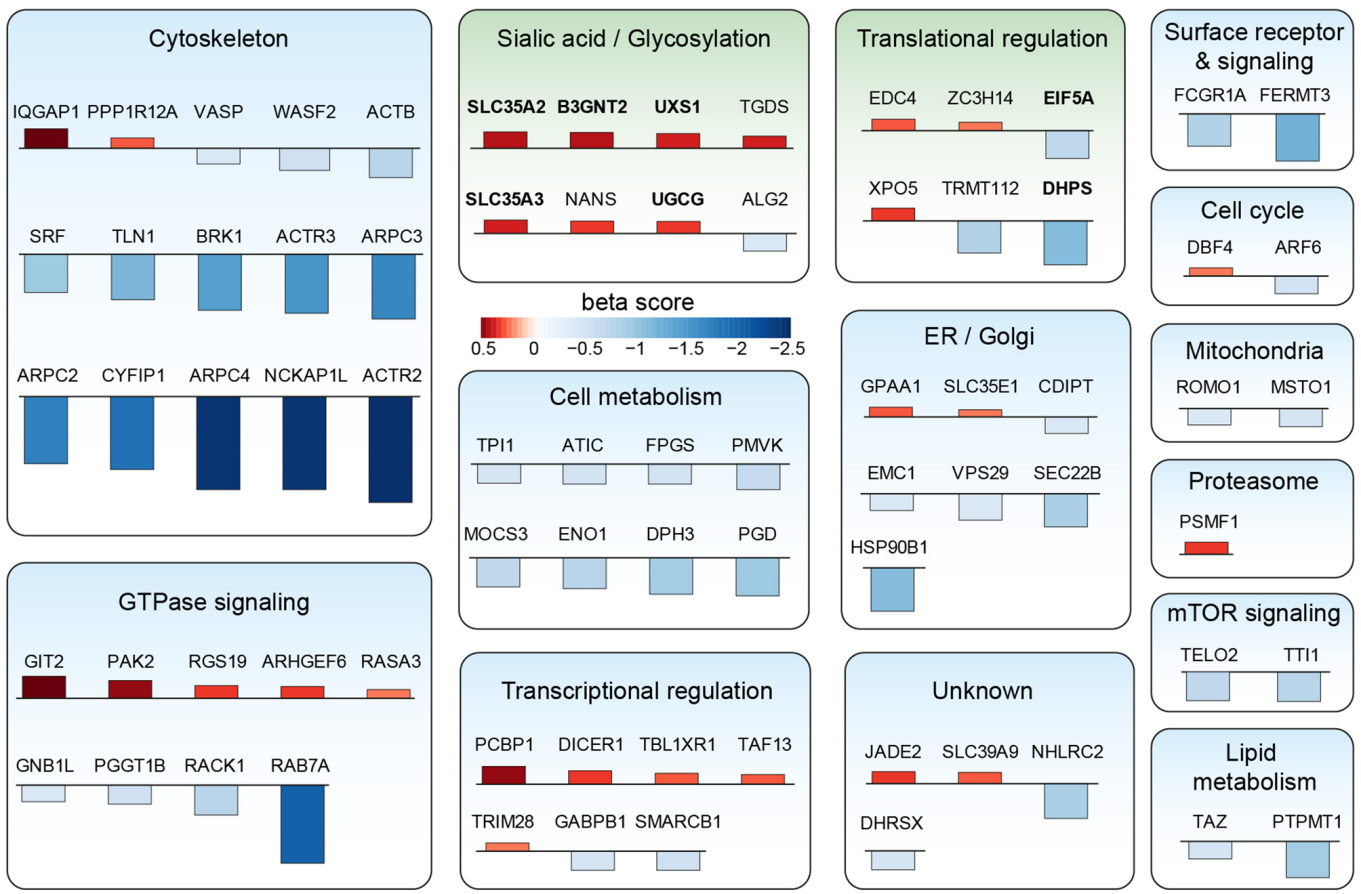
Functional classification of high confidence hits. The 75 high confidence hits were classified according to their function using GO terms and literature search. The direction of the bars indicates the sign of the beta score and the color and height indicates the value. Negative and positive beta values indicate genes whose knockout impairs or activates phagocytosis, respectively. Genes in bold are selected for single gene knockout validation.

MAGeCK MLE calculates beta scores for each gene, which is a measurement of the degree of selection similar to ‘log fold changes’ in differential expression analysis^23^. Comparing the beta values for the genes (Supplementary Tables S4 and S6) which had been investigated in both screens, we observed a high correlation with a Pearson correlation coefficient of 0.816. In both screens, the loss of the above described ARPC4 gene function impaired phagocytosis. Moreover, other members of the Arp2/3 complex (ACTR2, ACTR3, ARPC2 and ARPC3) and subunits of the WAVE complex (NCKAP1L, CYFIP1, BRK1), both known to regulate actin polymerization necessary for phagocytic cup formation, were also depleted in the phagocytosis high population. Additionally, RAB7A, a key regulator of endo-lysosomal trafficking and important for phagocytosis, was shown to be essential for bacterial phagocytosis (Fig. 2b). To identify high confidence hits out of both the genome-wide and the validation screen, only genes with an FDR ≤ 0.1 in both screens were selected. Finally, this resulted in 75 high confidence hits, with 28 gene knockouts activating phagocytosis and 47 inhibiting phagocytosis (Figs. 2a, 3).

Cellular localization analysis of the gene products for the 75 high confidence hits revealed them to be mostly located in the cytoplasm and nucleus, with 10 hits being associated with the plasma membrane (Figure S7a). The protein function of the hits is annotated mainly as enzymatic and other functions, for which a specific function is not known (Figure S7b). The top canonical pathways enriched are related to the actin cytoskeleton pathway, remodeling of epithelial adherens junctions, integrin signaling, Fcγ receptor-mediated phagocytosis in macrophages and monocytes, and various signaling pathways (Fig. 2c).

Next, we classified the hits to functional groups according to GO terms and literature search (Fig. 3). Most of the genes for which the gene knockout impairs phagocytosis are associated with the cytoskeleton in agreement with the canonical pathway analysis (Fig. 2c). Other larger groups are associated with the endoplasmic reticulum and Golgi apparatus, translational and transcriptional regulation, GTPase signaling, cell metabolism and glycosylation. In the latter group, five genes whose knockout activates phagocytosis are specifically involved in UDP glycosylation: Two members of the solute carrier family 35, SLC35A2 and SLC35A3, which transport UDP sugars from the cytosol to the lumen of the Golgi apparatus and the endoplasmic reticulum for glycosylation^26^, and B3GNT2, UXS1 and UGCG, which are enzymes of the UDP metabolism. Furthermore, two genes in the translational regulation cluster, EIF5A and DHPS show impaired phagocytosis when genetically inactivated. EIF5A is a translational elongation factor regulating the translation of a subset of mRNAs. Its activity depends on the modified amino acid hypusine^27^. There are two EIF5A isoforms present (EIF5A1 and EIF5A2), which in humans are the only proteins containing this amino acid modification^28^. Hypusine derives from a two-step conversion of lysine, catalyzed by the enzymes DHPS and DOHH.

**Figure 4:**
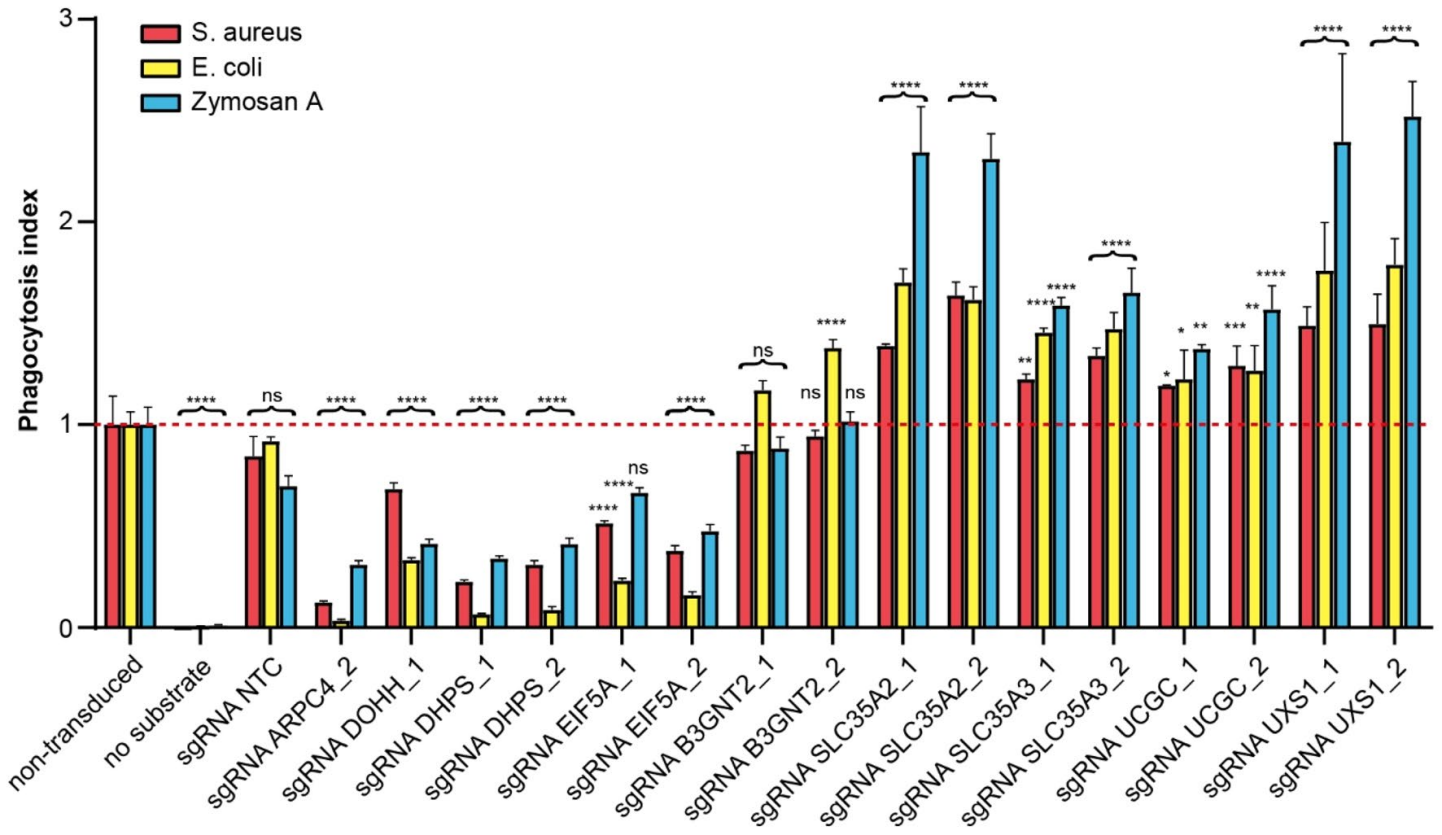
Validation of hits of EIF5A and related genes and genes involved in UDP-glycosylation pathway. THP-1 iCas9 cells were transduced with guides targeting indicated genes or with non-targeting control (NTC) and Cas9 expression was induced with dox. On day 12 after gene knockout, *S. aureus*, *E. coli* and zymosan A particles labeled with pHrodo red were added and phagocytosis was measured 1 h later using flow cytometry. Depicted is the mean phagocytosis index of live Thy1.1^+^ cells of 2–5 replicates ± SEM. The phagocytosis index is the mean fluorescence intensity normalized to non-transduced THP-1 cells. To test statistical significance, a one-way ANOVA corrected for multiple comparison according to Dunnett was performed. Ns: not significant, *p < 0.05, **p < 0.01, ***p < 0.001, ****p < 0.0001.

To validate and further characterize these genes, we created single gene knockouts with the two best-performing sgRNAs targeting each gene and performed phagocytosis assays with various substrates. DOHH, although neither a hit in the primary nor the secondary screen (FDR around 0.16 in both screens), was included in the validation experiment. Again, a non-targeting sgRNA was included as a negative control and an sgRNA targeting ARPC4 served as a positive control, inhibiting phagocytosis. Phagocytosis assays were performed in addition to *S. aureus* with *Escherichia (E.) coli* and zymosan A particles. As expected, compared to non-transduced cells, the non-targeting sgRNA showed no effect on phagocytosis of the 3 different substrates and the knockout of ARPC4 impaired phagocytosis of *S. aureus*, but also the phagocytosis of *E. coli* and zymosan A (3.2-fold, 8.1-fold and 28.8-fold for zymosan A, *S. aureus* und *E. coli*, respectively). The two sgRNAs targeting either EIF5A or DHPS inhibited phagocytosis of the various substrates, but to a different extent (*E. coli* > *S. aureus*/zymosan A). Knockout of DOHH impaired phagocytosis of the different substrate types with the weakest effects on *S. aureus* phagocytosis compared to the other experimental sgRNAs. This might explain why DOHH did not score in the two screens (Fig. 4). With exception of B3GNT2, knocking out the members of the UDP glycosylation pathway showed increased phagocytosis, and thus the screening hits were successfully confirmed by individual sgRNA transduction. The strongest effects were observed for the solute carrier SLC35A2 and the enzyme UXS1, increasing phagocytosis 2.4-fold and 2.5-fold, respectively. With respect to the three different particle types, phagocytosis of zymosan A was increased the most (up to more than twofold).

The fluorescence intensity of pHrodo red relies on the lysosomal acidification, hence, impaired acidification could result in reduced signal intensity. To evaluate whether the knockouts of the individual genes influence lysosomal acidification, KOs were stained with lysotracker. In none of the single gene knockouts an effect on lysosomal acidification compared to the non-targeting control sgRNA was observed, with exception of the sgRNA_2 targeting EIF5A. This knockout resulted in a small reduction of the lysotracker signal indicating a weak effect of the EIF5A KO on lysosomal acidification. Compared to the effect on phagocytosis, the changes in lysosomal pH are rather small (Figure S8a). Treatment of the various KO cells with 100 nM bafilomycin A, an inhibitor of the lysosomal V-type proton ATPase^29,30^ led to a reduction of the lysosomal pH in all KO cells, showing no additional/synergistic effects of the different KOs on bafilomycin A induced lysosomal pH changes. To further discriminate phagocytosis from other forms of endocytosis, we assessed the uptake of transferrin and dextran as models for clathrin-mediated and clathrin-independent endocytosis assays^31^. We used 10 kDa dextran allowing for efficient uptake by macropinocytosis^32,33^. The endocytic uptake of transferrin and dextran was not affected by the single gene knockouts except for DOHH for which a 29% reduction of the transferrin uptake was observed (Figure S8b, c). Compared to the effects of a DOHH knockout on phagocytosis, the reduced effect of transferrin uptake is less pronounced. Whereas for phagocytosis actin dependent cytoskeleton rearrangements are indispensable, for other forms of endocytosis actin filament reorganization is less important^31^. Therefore, it is not surprising that cytochalasin D, which inhibits actin polymerization does not block endocytosis of dextran and only reduce endocytosis of transferrin by about 20% irrespective of the gene knockout. Phagocytosis of *E. coli* particles in the presence of cytochalasin D could be completely blocked (Figure S8d).

## Discussion

Phagocytosis is an integral mechanism of the immune system of which dysregulation is associated with various diseases. We performed pooled CRISPR screens to identify genes regulating phagocytosis of the gram-positive bacterium *S. aureus*., and further confirmed the hits by individual sgRNA transduction experiments and testing additional phagocytosis substrates (*E. coli* and zymosan A particles). We used commercially available, well described particles derived from inactivated bacteria or yeast zymosan A.

Over the last years, pooled CRISPR-Cas9 screening technology has been optimized to become a robust tool to study gene function at both the cellular^16,21,34,35^ and the organismal^36^ level. Genome-wide CRISPR screens have been successfully applied to delineate biological pathways^16,34,35,37^ and to identify candidate drug targets^38^. Although genome-wide sgRNA libraries enable the systematic perturbation of the entire coding or regulatory genome, adapting CRISPR screens for probing gene functions in specific cellular processes requires the development of cell-based assays specific to the pathway or activity of interest. For example, recently described screens used either an engineered reporter to study autophagy by FACS analysis^37^ or phagocytosis by MACS enrichment^16^. Here, we describe the successful combination of our recently published FACS-based phagocytosis assay^20^ with the pooled CRISPR-Cas9 screening technology to elucidate known and unknown regulators of phagocytosis at the genome level. The identification and validation of hits depended on the increase and decrease of pHrodo red signal intensity. After internalization endosomes are fused to lysosomes leading to acidification which increases the pHrodo red signal intensity. Using pH-sensitive lysotracker, we verified that acidification is not impaired in single gene knockouts of the identified hits.

Recently, a systematic investigation of genetic regulators of phagocytosis was published by Haney and co-workers^16^. They performed pooled CRISPR screens in macrophage-differentiated U937 cells, a pro-monocytic cell line, using various particle types for phagocytosis including beads, IgG- or C3a-opsonized apoptotic cells, myelin and zymosan. By contrast, we used the human monocytic THP-1 cell line and a bacterial phagocytosis substrate (*S. aureus*) in our pooled screens. We confirm and extend their results with respect to the essential role of the actin cytoskeleton regulating Arp2/3 and WAVE complexes in phagocytosis. As already described for U937 phagocytosis, loss of different subunits in these complexes is detrimental for *S. aureus* phagocytosis in THP-1 cells. Interestingly, a similar gene set has lately been shown to inhibit the uptake of *Salmonella* by macrophage differentiated THP-1 cells^17^. RAB7A, another top scoring hit in our screens has been shown to be essential for phagocytosis. RAB7A acts more downstream in phagosome maturation and is essential for the transition from early to late phagosome^39^.

We were surprised to find and confirm genes involved in glycosylation. The knockout of USX1, UGCG, SLC35A2 and SLC35A3 increased phagocytosis not only of *S. aureus*, but also *E. coli* and zymosan A. UXS1 catalyzes the conversion of UDP-glucuronic acid to UDP-xylose, which is a substrate for three glycosylation processes involved in the biosynthesis of glycosaminoglycans, O-glycans and dystroglycans^40^. UGCG generates glucosylceramide, which is the precursor for all glycosphingolipids^41^. SLC35A2 and SLC35A3 are transmembrane transporters for UDP-galactose and UDP-N-acetylglucosamine, respectively. They transport UDP sugars from the cytosol to the lumen of the Golgi apparatus and the endoplasmic reticulum for glycosylation^42^. The solute carriers SLC35A2, SLC35A3 and SLC35A4 together with N-acetyl-glucosaminyltransferases self-assemble into multi-enzyme/multi-transporter complexes to facilitate the synthesis of complex N-glycans^43^. To our knowledge, only a few reports showing an effect of protein glycosylation in the phagocyte on its rate of phagocytosis are available, e.g. O-glycosylation of the cell surface protein C1qRp enhances both FcR- and CR1-mediated phagocytosis^44,45^. By contrast, many reports demonstrate effects of the glycosylation status of the phagocytosed particle on phagocytosis. For instance, it was shown that O-glycosylation of the bacterial cell wall^46^ and decorin-coating of collagen fibers^47^ influences their phagocytosis. Moreover, antibodies and their corresponding Fc receptors are also highly glycosylated and the correct glycosylation is important for binding of Fc receptors to antibodies, which in turn is essential for antibody-dependent phagocytosis^48^. However, currently we do not know whether and how the knockout of the described genes influenced the protein glycosylation pattern in our THP-1 cell line. In addition, B3GNT2 was identified in our screens. However, the single knockouts of B3GNT2 showed no effect on phagocytosis of *S. aureus* and zymosan A, but an increase in phagocytosis of *E. coli*. B3GNT2 is a polylactosamine synthase that synthesizes a backbone structure of carbohydrate structures onto glycoproteins. It is expressed in murine macrophages, and B3GNT2-deficient mice show dysregulated activation of macrophages with elevated levels of CD14 expression and an enhanced response to endotoxin^49^. Together, all five genes are members of the cellular glycosylation machinery and their knockouts increased phagocytosis. However, a phagocytosis phenotype has not been described for either of them before, and further research is required to understand their role in the process of phagocytosis, which is beyond the scope of this study.

We identified two additional genes with no described link to phagocytosis, EIF5A and DHPS. Together with DOHH, DHPS is required for hypusination of EIF5A. The effect of the three genes on phagocytosis was tested individually and we could confirm that their knockout inhibits phagocytosis. EIF5A is a translational elongation factor and the only human protein which undergoes hypusination^50^. Hypusination is a posttranslational modification of lysine, which is catalyzed by the gene products of DHPS and DOHH^28^. EIF5A and the polyamine synthesis pathway regulate oxidative phosphorylation. Macrophages stimulated with IL-4 but not with LPS plus IFN-γ upregulate the hypusinated form of eIF5A^51^. Moreover, genetic ablation of Eif5a, Dhps or Dohh reduced the expression of CD206 and CD301 in murine macrophages, which are markers of alternative activation^51^. Furthermore, it was shown that the proteins Ldp17 and Vrp1, which are related to actin cytoskeleton organization, are nearly absent in *Saccharomyces cerevisiae* temperature-sensitive mutants of eIF5A^50^. This might explain why we observe a markedly reduced phagocytosis of *S. aureus*, zymosan A and especially *E. coli* particles in DHPS, DOHH and EIF5A knockouts.

To confirm that the genes identified in our genetic screen are phagocytosis specific and no general inhibitors of endocytosis, we tested there knockout effects in models for clathrin-dependent and -independent endocytosis^52^, i.e. transferrin and dextran uptake, respectively. Neither dextran, nor transferrin uptake was substantially affected by any one of the single knock outs. However, DOHH knockout showed 29% reduction of transferrin endocytosis indicating a more general impairment of uptake.

In summary, we successfully performed a CRISPR-Cas9 screen to identify yet unknown genes involved in phagocytosis. Among these, our screen reveals several components in glycosylation and hypusination pathways, which have not been implicated previously as regulators of phagocytosis. Even though classical receptors for foreign particles including opsonic receptors were not among the top hits, the targets identified might impact their expression or might alter their binding affinity by modifying the glycosylation pattern. Interestingly, single-gene knockouts of selected hits demonstrated that not only the phagocytosis of *S. aureus* particles but also of *E. coli* and zymosan A particles were affected. This indicates that many of the identified hits are either ubiquitously involved in phagocytosis or are at least important for the uptake of both bacteria and yeast. This study broadens the spectrum of genetic regulators of phagocytosis. However, their exact molecular mechanism in the process of phagocytosis and their regulation and role in human disease processes needs to be addressed in future studies.

## Methods

### THP-1 cell culture and engineering

THP-1 (TIB-202 from ATCC) cells were cultured in RPMI1640 + Glutamax-I Medium (Gibco, 61870-010) with 10% FCS (Invitrogen, 50064), 1% sodium pyruvate (Gibco, 11360-070) and 1% penicillin/streptomycin (Gibco, 15140-122) at 37 °C and 5% CO_2_. Cells were passaged twice per week and kept at a cell concentration of below 2E06/mL. To derive Tet-inducible Cas9-GFP (THP-1 iCas9) cells, THP-1 cells were sequentially transduced with pLenti-EF1A-rtTA3-IRES-EcoRec-PGK-Puro and pLenti_TRE3G-Cas9-P2A-GFP. After 2 days of dox treatment (500 ng/mL, Sigma-Aldrich, D9891), Cas9-GFP-inducing single-cell clones were isolated using FACS, expanded, and tested for homogenous Cas9-GFP induction. To evaluate the efficacy of CRISPR/Cas9 editing, individual clones were transduced with a lentiviral vector co-expressing an sgRNA targeting human CD46 and mCherry (pLenti-U6-sgRNA.iT-EF1as-mCherry). Following 5 days of Cas9-induction using dox (100 ng/mL), CD46 expression was characterized in sgRNA/mCherry + cells using flow cytometry. To further characterize the selected clone, THP-1 iCas9 cells were transduced with a pool of lentiviral sgETN vectors^21^ containing sgRNAs targeting essential genes (TIMELESS, WDHD1, RAD21, SMC3, PLK1) or a non-targeting control. Co-expression of murine Thy1.1 from sgETN was used to enrich transduced cells 2 days after lentiviral infection applying the MACS technology in combination with CD90.1 microbeads (Miltenyi, 130-094-523). Enriched cells were stimulated with 100 ng/mL dox on day 2, 3 and 7 post transduction for Cas9 induction. Cells were cultured for up to 14 days. On day 6, 9, 11 and 14 post transduction cells were counted and analyzed for Thy1.1 expression by flow cytometry. To this end, cells were stained with Thy1.1-APC (Abcam, ab95810) and the fraction of Thy1.1-positive cells was determined.

### Genome-wide sgRNA library and generation of focused sgRNA pools

Design, construction, and basic performance of the genome-wide sgRNA library has been described in Michlits et al.^21^. To generate focused sgRNA pools for validation screens, oligo pools were obtained from Twist Bioscience and amplified by PCR using the Q5 Hot Start High-Fidelity DNA Polymerase (NEB). The amplicons and the sgETN vector were restricted with Esp3I (Thermo Fisher Scientific), the plasmid vector was purified by agarose gel electrophoresis and the digested sgRNAs were purified by ethanol precipitation. For the ligation, a total of 2 µg backbone was used at a vector to insert ratio of ~ 1:10. Ligation was performed at 16 °C overnight using T4 DNA Ligase (NEB). The ligation reaction was purified using phenol extraction with subsequent ethanol precipitation. The precipitated ligation reaction was dissolved in 15 µL TE buffer and 4 µL were used for transformation of MegaX DH10B T1 cells (Thermo Fisher Scientific). Bacteria were plated on LB agar dishes containing ampicillin and incubated at 37 °C overnight. The next day all bacteria were scratched off the plates and cultivated in 1 l LB medium (with ampicillin) for about 6 h. Bacteria were harvested, and plasmid DNA was prepared using the NucleoBond Xtra Maxi EF (Macherey Nagel) according to the manufacturer’s recommendations. Cloning of single sgRNA (Supplementary Table S1) was performed as described in Datlinger et al.^53^. Briefly, two reverse complementary oligos with overhangs were hybridized, phosphorylated and cloned into a pre-cut vector backbone.

### Generation and quantification of lentiviral particle

Lentiviral vectors were produced using MISSION Lentiviral Packaging Mix (Sigma-Aldrich), PEI Max (Polysciences, Inc.) and Lenti X-293T cells (Clontech Laboratories, Inc) according to the manufacturer’s recommendations. Virus supernatants were collected on day 3 and 4 after transfection and concentrated 100× using the PEG-it Virus Precipitation Solution (System Biosciences). For quantification, RNA was extracted using Viral Xpress (Merck), DNA was digested using RQI RNase-free DNase (Promega), and cDNA was generated with High-Capacity cDNA Reverse Transcription Kit (Thermo Fisher Scientific). Viral genomes were determined either by qPCR or ddPCR with TaqMan Assay on Demand targeting a WPRE element in the lentiviral vector using the following primers: 5′-GCATTGCCACCACCTGTCA-3′, 5′-TCCGCCGTGGCAATAGG-3′ and a FAM probe: 5′-CTTTCCGGGACTTTCG-3′ (Thermo Fisher Scientific).

### CRISPR screen (transduction, MACS purification, Dox induction, FACS)

THP-1 iCas9 cells were transduced with lentiviral particles using spinfection. To this end, THP-1 cells were transferred into 6-well plates, virus was added and plates were centrifuged at 1000×*g* for 20 min. Two days (genome-wide screen) or five days later (validation screen), transduced cells were MACS enriched using CD90.1 MicroBeads (Miltenyi Biotec). Cells were stimulated with dox (1 µg/mL, Sigma-Aldrich) on two consecutive days or left untreated for baseline samples. Cells were split twice per week until phagocytosis assay (11 and 12 days after dox stimulation for genome-wide and validation screen, respectively). For the phagocytosis assay the medium was changed to serum-free X-Vivo 10 medium (Biozym). *S. aureus* pHrodo red particles (Thermo Fisher Scientific) were added and cells were incubated for 1 h at 37 °C and 5% CO_2_. After one hour, cells were placed on ice to stop further phagocytosis. Cells were stained with anti-Thy1.1-APC (clone: HIS51, eBioScience) and DAPI (Thermo Fisher Scientific), and sorted on a BD FACSAria Fusion (BD Biosciences).

### DNA isolation

FACS-enriched or baseline cells were collected by centrifugation, washed with PBS and a maximum of 5E6 cells were resuspended in 350 µL DNA extraction buffer (10 mM Tris-HCl, 150 mM NaCl, 10 m M EDTA, pH 8.0). After addition of each 3.5 µL 10% SDS (Invitrogen) and 20 mg/mL proteinase K (Sigma), the suspension was incubated at 55 °C overnight. Proteinase K was heat inactivated at 95 °C for 10 min, followed by incubation with 10 mg/mL RNase A at 37 °C for 30 min. The mixture was transferred to a QIAshredder (Qiagen) and spun at 20,000 g for 2 min. Phase Lock heavy tubes (Quantabio) were spun and 350 µL phenol/chloroform/isoamyl alcohol (Roth) was added. Samples were mixed by shaking. Samples were spun at 20,000 g for 8 min. The upper phase was chloroform extracted twice with 350 µL chloroform, and DNA was ethanol-precipitated (35 µL 3 M Na-acetate pH 5.2 and 1050 µL absolute ethanol) at − 20 °C overnight. Samples were centrifuged at 20,000×*g* for 30 min. The DNA pellet was washed twice with 70% ethanol, dried and resuspended in 50 µL buffer EB (Qiagen).

### Sequencing

The sequencing libraries were generated using a two-step PCR. The first PCR amplifies the target specific region and adds adapter sequences used as template for the second PCR. From each sample, the complete DNA was used for the sequencing library preparation. In each 100 µL PCR 1 µg of template was amplified using Q5 Hot Start High-Fidelity 2X Master Mix (NEB), additional 2 mM MgCl_2_ and a pool of forward and reverse primers containing 2 to 8 nucleotide staggers and the adapter sequence (Supplementary Table S2). Amplicons were purified using 0.8 × Agencourt AMPure XP beads (Beckman Coulter) and the MagMax (Thermo Fisher Scientific). Purified amplicons from the same sample were pooled and 20 ng was used for the second PCR using NEBNext Multiplex Oligos for Illumina and NEBNext Ultra II Q5 Master Mix (both NEB). The final sequencing libraries were cleaned up twice as describe before and quality controlled with the Fragment Analyzer (AATI). Sequencing was performed on Next-Seq 550 (Illumina) with High-Output and 150 bp single-end mode.

### Endocytosis assay of single gene knockouts

THP-1 cells were either treated with 10 µM cytochalasin D (PHZ1063, Gibco) at 37 °C and 5% CO2 for 30 min or left untreated. *S. aureus*, *E. coli*, zymosan A, dextran (25 µg/mL, 10 kDa, P10361) or transferrin particles conjugated with pHrodo red were purchased from Thermo Fisher Scientific. Particles were added to cells and cells were incubated at 37 °C and 5% CO2 for 1 h. Of note, cytochalasin D was not removed before addition of particles and the final concentration during particle uptake was 7.5 µM. After the incubation, cells were placed on ice to stop further phagocytosis. Cells were stained with anti-Thy1.1-APC (clone: HIS51, eBioScience) and DAPI (Thermo Fisher Scientific), and analyzed on BD LSRFortessa X20 (BD Biosciences). Cells were gated based on FSC/SSC, singlets, live (DAPI-) and Thy1.1^+^ cells using FlowJo V10.5.3 or FACS DIVA (Becton, Dickinson & Company). The mean fluorescence intensity (MFI) was determined and normalized to the MFI of non-transduced cells.

### High content imaging

THP-1 cells were either treated with 100 nM bafilomycin A1 (Sigma) at 37 °C and 5% CO_2_ for 1 h or left untreated, followed by an 1 h incubation with LysoTracker Deep Red (Molecular Probes) in a 384-well view plate. Pictures were taken using the Opera Phenix confocal microscope (PerkinElmer) and the analysis was done in Columbus software version 2.8.2.1205.

### Data analysis

Sequences upstream and downstream of the guide sequence were trimmed off with Cutadapt (V1.17)^54^. Reads were mapped to the guide reference using MAGeCK-VISPR (V0.5.3)^23^. Counts were normalized to the median count. Samples with increased phagocytosis were compared to samples with reduced phagocytosis using the MLE algorithm in MAGeCK (V0.5.6)^22^. Further analysis, e.g. filtering, comparing, plotting, was performed in R studio (V1.2.1335-1, R 3.5.2). One-way ANOVA corrected for multiple comparison according to Dunnett was calculated with GraphPad Prism (version 8.0.0).

## Supplementary Information

Supplementary Information 1.

Supplementary Information 2.

## Data Availability

All data generated or analyzed during this study are included in this published article (and its Supplementary Information files).

## Abbreviations

sgRNA: Single-guide RNA
Ig: Immunoglobulin
MACS: Magnetic-activated cell sorting
FDR: False discovery rate
