# Supplementary material for: A genome-wide CRISPR/Cas9 screen to identify phagocytosis modulators in monocytic THP-1 cells
Source: Sci Rep. 2021 Jun 21;11:12973. doi: 10.1038/s41598-021-92332-7 (PMC8217514; doi:10.1038/s41598-021-92332-7)
Supplement: Supplementary file 1 — Supplementary Information 1. [file 41598_2021_92332_MOESM1_ESM.docx]

A genome-wide CRISPR/Cas9 screen to identify phagocytosis modulators in monocytic THP-1 cells

Benjamin Lindner^1*^, Eva Martin^1^, Monika Steininger^2^, Aleksandra Bundalo^2^, Martin Lenter^1^, Johannes Zuber^2,3^, Michael Schuler^1^

**Authors’ affiliation:**

^1^: Drug Discovery Science, Boehringer Ingelheim Pharma GmbH & Co. KG, 88397 Biberach an der Riss, Germany

^2^: Research Institute of Molecular Pathology (IMP), Vienna BioCenter (VBC), 1030 Vienna, Austria.

^3^: Medical University of Vienna, Vienna BioCenter (VBC), 1030 Vienna, Austria

*: Corresponding Author:

[benjamin.lindner@boehringer-ingelheim.com](mailto:benjamin.lindner@boehringer-ingelheim.com)


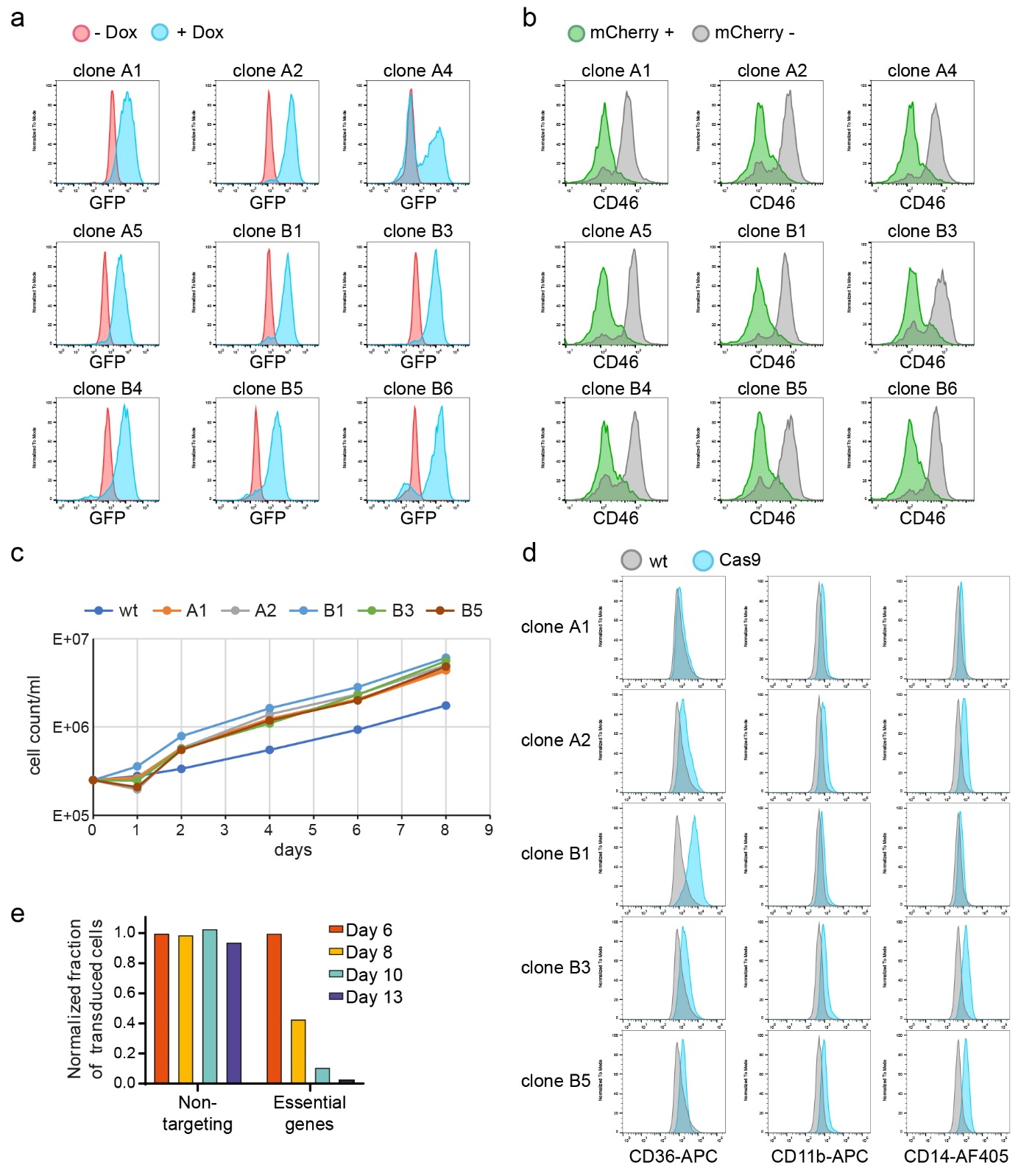


**Figure S1: Validation of Cas9 functionality in THP-1 iCas9 cells**. THP-1 iCas9 clones were treated with dox to assess co-induction of GFP with Cas9 by flow cytometry, resulting in a shift in GFP-signal (+ dox) (**a**). THP- iCas9 cells were transduced with a lentiviral vector co-expressing an sgRNA targeting CD46 and mCherry, at an infection rate of about 50% of the cells. CD46 surface expression was assessed by flow cytometry and compared between the sgRNA-harboring population (mCherry+) and the non-transduced (mCherry-) population. (**b**). The growth rate of five THP-1 iCas9 clones was measured for 8 days by determining the cell count of the clones compared to wt THP-1 cells (**c**) and the surface expression of the monocytic markers CD35, CD11b and CD14 was assessed by flow cytometry (**d**). Clone A2 was transduced with a lentiviral vector containing sgRNAs targeting essential genes or a non-targeting control. On day 3 after transduction, Cas9 expression was induced with addition of dox leading to CRISPR mediated gene knockout. On day 6, 8, 10 and 13 post transduction, cells were counted and analyzed by flow cytometry. The ratios of transduced cells (expressing murine Thy1.1) and non-transduced cells were calculated and normalized to the ratio of day 6 (**e**).

**
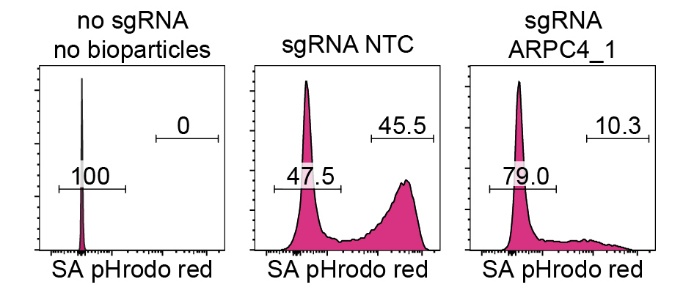
**

**Figure S2: Phagocytosis of *S. aureus* particles can be blocked by CRISPR-mediated knockout of ARPC4.** THP-1 iCas9 cells were transduced with an sgRNA targeting ARPC4 or a non-targeting sgRNA, enriched by MACS and Cas9 was induced with dox for two consecutive days. 14 days after transduction, *S. aureus* particles labeled with pHrodo red was added to the cells. After 60 min, cells were analyzed for their pHrodo signal. Cells without addition of *S. aureus* showed no pHrodo red signal. THP-1 cells transduced with non-targeting sgRNAs show a similar distribution of phagocytosing versus non-phagocytosing cells. Transduction with a sgRNA targeting ARPC4 reduced the percentage of phagocytosing cell approximately 4-fold.


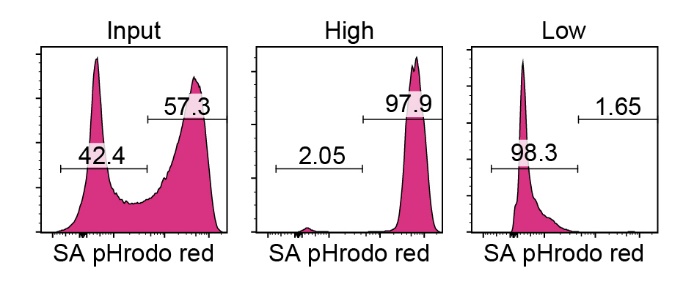


**Figure S3: Sorting of phagocytosing and non-phagocytosing THP-1 cells.** *S. aureus* particles labeled with pHrodo red was added to THP-1 iCas9 cells. After 60 min, cells were sorted by FACS according to their fluorescence intensity in phagocytosing and non-phagocytosing populations. Before the sort, the mixed cell population consisted of about 50 % phagocytic active and inactive cells. After sorting, a purity of about 98 % was achieved (middle and right panel).


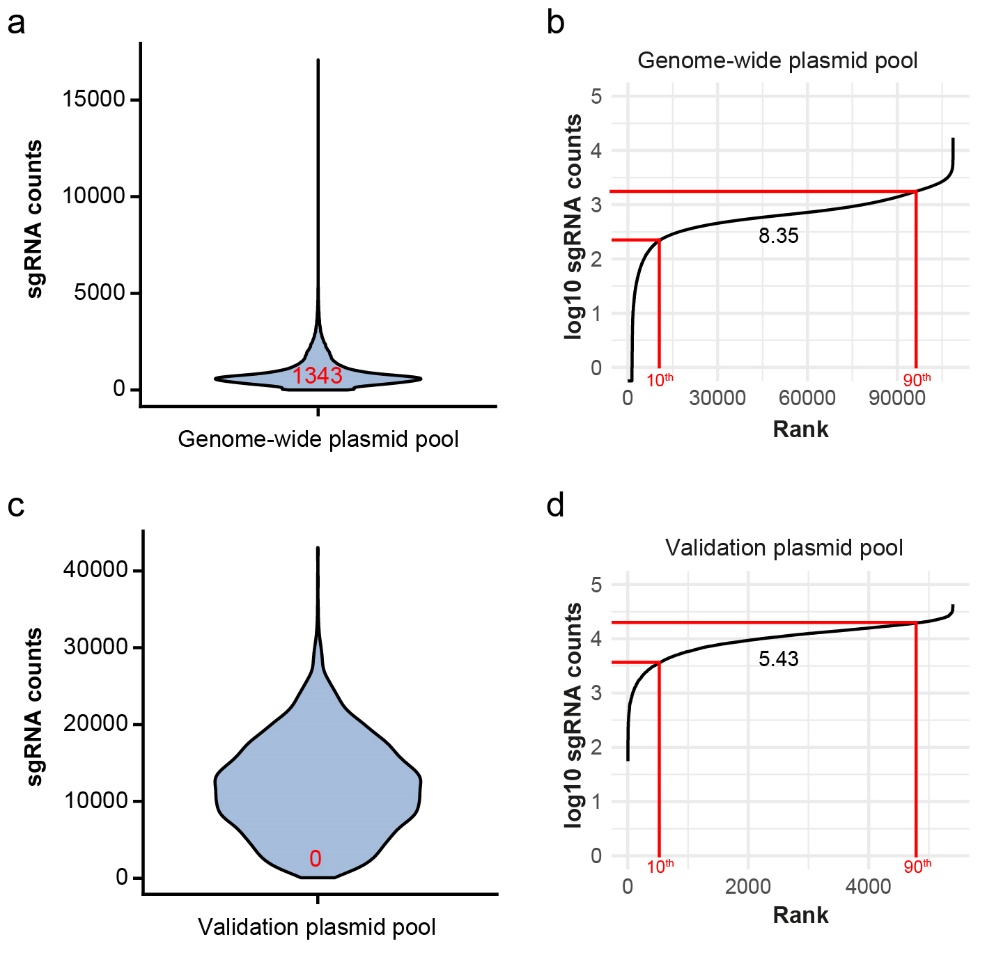


**Figure S4: Quality control of plasmid pools.** Amplicon libraries of plasmid pools were generated and sequenced with NextSeq 550. Raw reads were analyzed with MAGeCK-VISPR. The sgRNA distribution of reads for the genome-wide and validation pools are depicted as violin plots and the number of missing sgRNA (i.e. having a count of 0) are highlighted in red (**a**, **c**). The sgRNA counts are ranked and the 10^th^ and 90^th^ percentile are drawn as red lines and the ratio of the 90^th^ to the 10^th^ percentile was calculated (**b**, **d**).


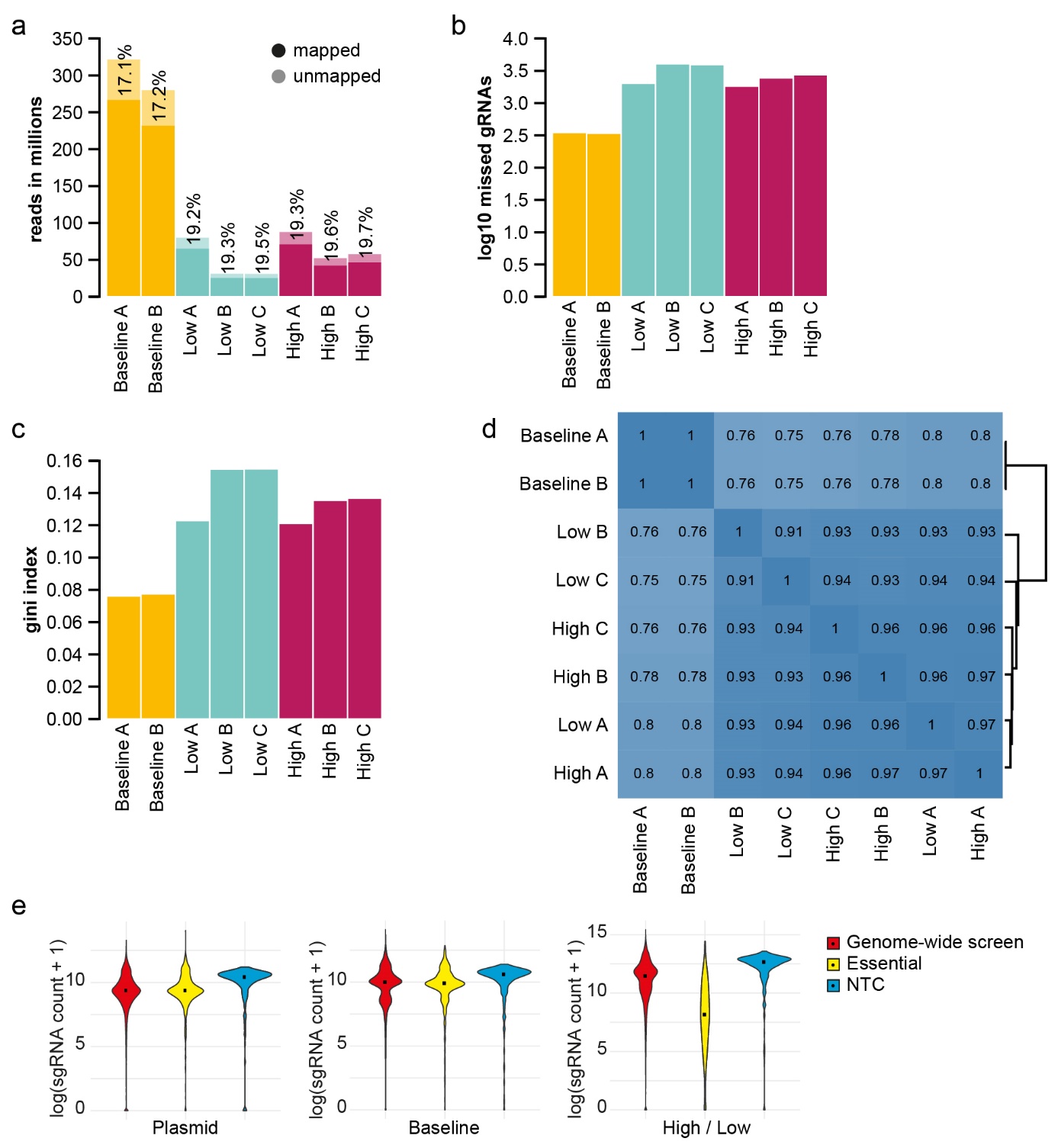


**Figure S5: MAGeCK-VISPR analysis of the genome-wide screen.** Trimmed reads were analyzed with MAGeCK-VISPR with normalization to median counts. For each sample, the mapping rates (**a**), missing sgRNAs (**b**), and the gini index were calculated (**c**). The correlation of samples is given in **d**. In **e**, the sgRNA counts were grouped into non-targeting controls (NTC), essential genes, and genome-wide screen and the distribution of the sgRNA pools of the plasmid pool, baseline samples and sorted cell populations (high / low: phagocytosing and non-phagocytosing) are shown.


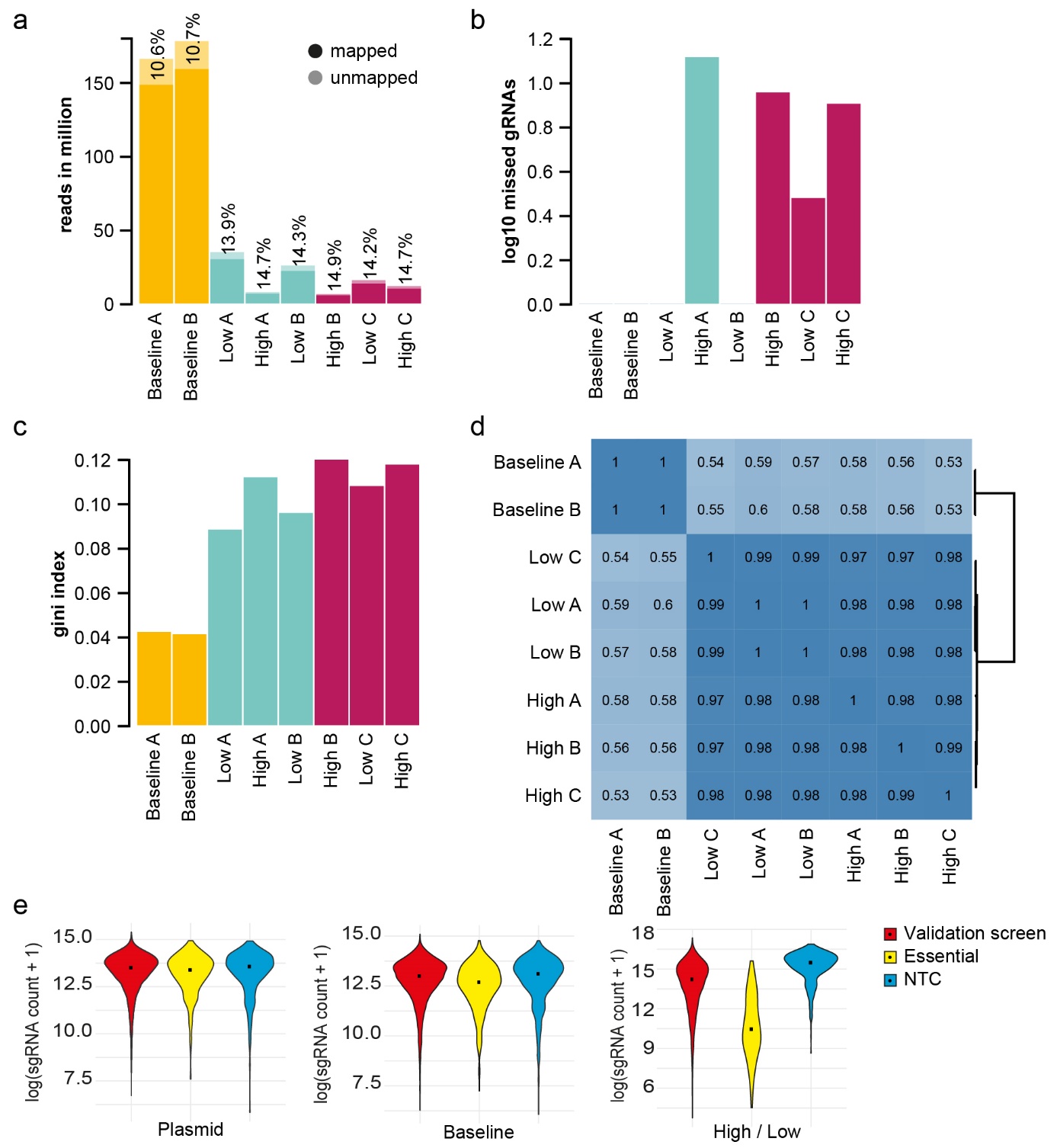


**Figure S6: MAGeCK-VISPR analysis of the validation screen.** Trimmed reads were analyzed with MAGeCK-VISPR with normalization to median counts. For each sample the mapping rates (**a**), missed sgRNAs (**b**), and the gini index were calculated (**c**). In **a**) the frequencies of unmapped reads are given. The correlation of samples is given in **d**. **e**, The sgRNA counts were grouped into non-targeting controls (NTC), essential genes, and validation screen and the distribution of the sgRNA pools of the plasmid pool, baseline samples and sorted cell populations (high/low: phagocytosing and non-phagocytosing) are depicted.


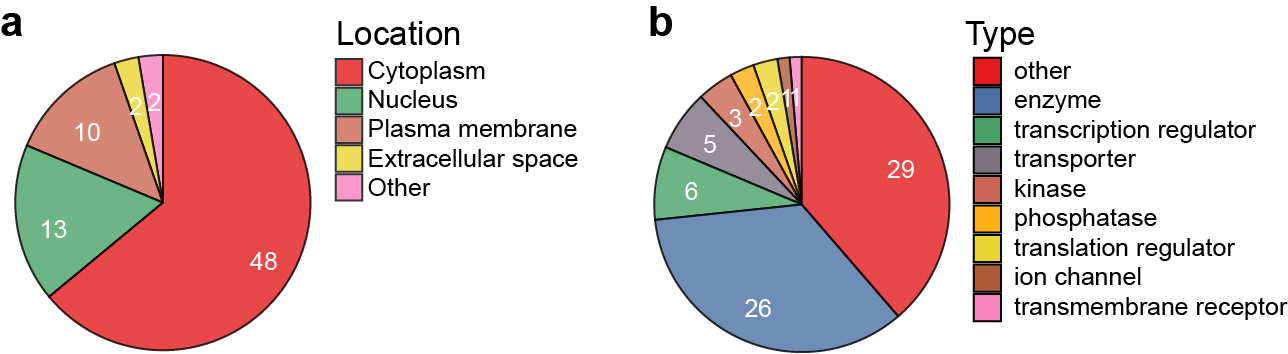


**Figure S7: Location and type of high confidence hits** Genes were annotated with Ingenuity Pathway Analysis (IPA) and sorted according to location (**a**) and type (**b**).

**
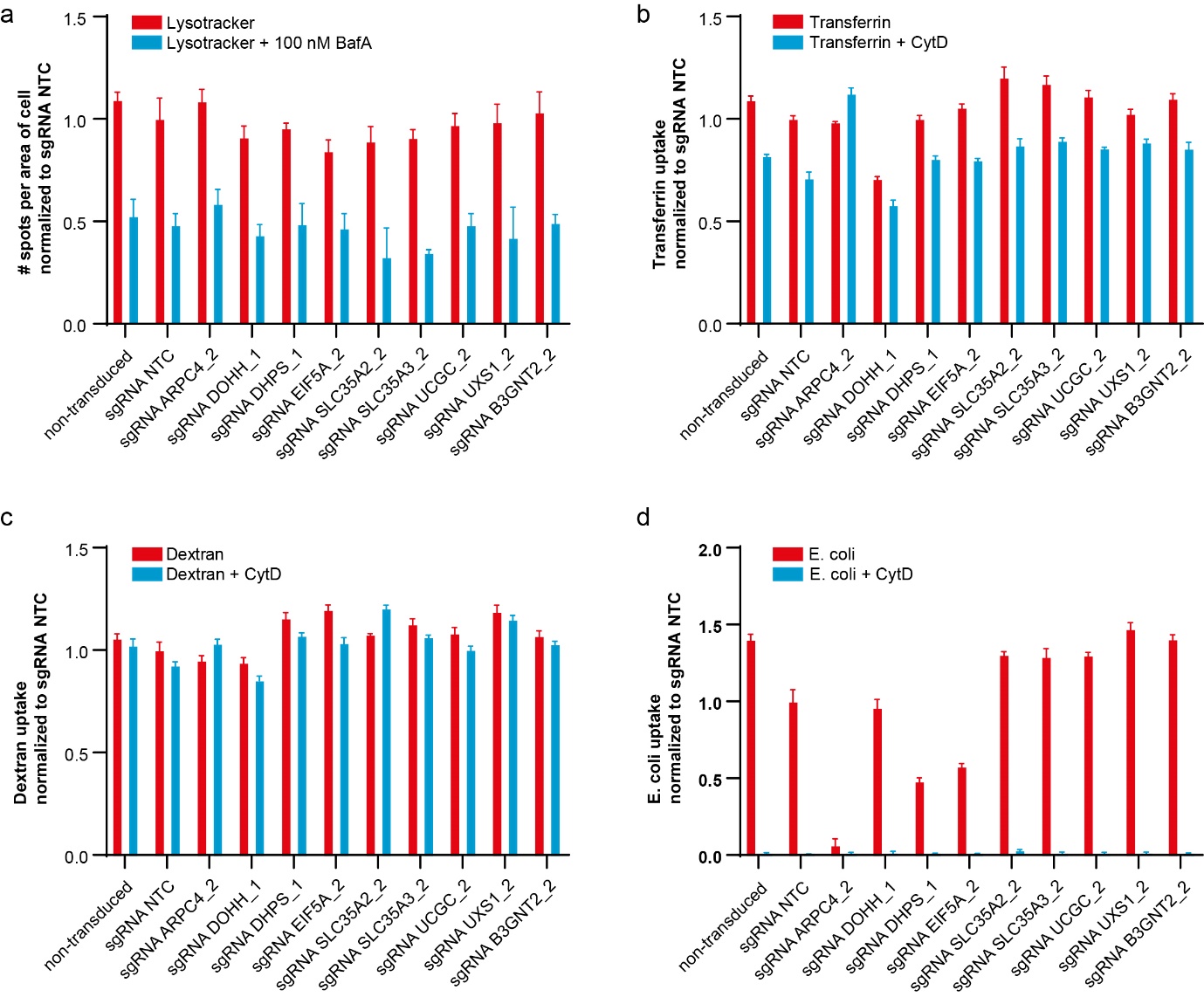
**

**Figure S8: Individual gene knockouts show normal acidification of lysosomes and endocytic capabilities. a**) sgRNA infected THP-1-Cas9 were either treated of not with bafilomycin A (BafA), before incubation with lysotracker After 1 h, lysosomal staining was as spots per area of cell. All data are normalized to the NTC-sgRNA. B-D) sgRNA transduced and non-transduced cells were either preincubated or not with 10 µM cytochalasin D (CytD) for 30 min. Then, pHrodo-red-labeled **b**) transferrin, **c**) dextran (10 kDa) or **d**) *E. coli* particles were added for 1 h of incubation, after which substrate uptake was measured by flow cytometry. Mean fluorescence intensities were normalized to NTC-sgRNA, the mean ± SD for n=3-4 technical replicates.

**Supplementary tables**

**Table S1: List of sgRNAs cloned in sgETN.**

| **ID** | **Target gene** | **Internal** | **Guide sequence** |
| --- | --- | --- | --- |
| sgRNA TIMELESS | TIMELESS | 8914_41 | GCTCATACAAGGTTTCACTG |
| sgRNA WDHD1 | WDHD1 | 11169_28 | GTGGAAACCAAAGACTGCA |
| sgRNA RAD21 | RAD21 | 5885_2 | GATCGTGAGATAATGAGAGA |
| sgRNA SMC3 | SMC3 | 9126_9 | GGATGCAAGAGATAAAATGG |
| sgRNA PLK1 | PLK1 | 5347_26 | GAGGTGCTGAGCAAGAAA |
| sgRNA NTC | N/A |  | GTAGCGAACGTGTCCGGCGT |
| sgRNA ARPC4_1 | ARPC4 | 10093_2_fillup | GGAACTCTTCAGCCACAATG |
| sgRNA ARPC4_2 | ARPC4 | 10093_6_fillup | GGAGAAGTTCTCCAGGCAG |
| sgRNA DHPS_1 | DHPS | 1725_13 | GTGCTGCACAAGGTAGCGAA |
| sgRNA DHPS_2 | DHPS | 1725_8 | GGGCGCCAGGCACTTGATG |
| sgRNA DOHH | DOHH | 83475_1 | GGGCGGAATCGTCATCGA |
| sgRNA EIF5A_1 | EIF5A | 1984_3_fillup | GGACAGCGGGGAGGTACGAG |
| sgRNA EIF5A_2 | EIF5A | 1984_4 | GCACCAGGACAGCCTCATGG |
| sgRNA B3GNT2_1 | B3GNT2 | 10678_12 | GAGCATGCTGACCAACCAGA |
| sgRNA B3GNT2_2 | B3GNT2 | 10678_21 | GGGGCCAAGAAAGCAACGCA |
| sgRNA SLC35A2_1 | SLC35A2 | 7355_2 | GGATGTCCCCAACATCAAA |
| sgRNA SLC35A2_2 | SLC35A2 | 7355_5 | GCAAGGTGTAGATGAGAG |
| sgRNA SLC35A3_1 | SLC35A3 | 23443_5 | GCAAGTTTAAGTGTTTCCAT |
| sgRNA SLC35A3_2 | SLC35A3 | 23443_6 | GAAAGACCAAAATTCCCA |
| sgRNA UGCG_1 | UGCG | 7357_3 | GATTACACCTCAACAAGA |
| sgRNA UGCG_2 | UGCG | 7357_8 | GATGTATGTAAGAAGCTTCT |
| sgRNA UXS1_1 | UXS1 | 80146_5 | GGGAGGCTGGAGATGCCAGA |
| sgRNA UXS1_2 | UXS1 | 80146_3 | GTTTAATGTCCCAATCGTAT |

**Table S2: List of fusion primers used for the amplification of integrated sgRNAs.** The 7 forward and reverse primers were used as a pool to increase the base diversity for sequencing.

| **Name** | **Adapter sequence** | **Stagger** | **Target specific sequence** |
| --- | --- | --- | --- |
| sgScreen_Fusion_F1.1 | ACACTCTTTCCCTACACGACGCTCTTCCGATCT | NN | AAAATGGACTATCATATGCTTACCG |
| sgScreen_Fusion_F1.2 | ACACTCTTTCCCTACACGACGCTCTTCCGATCT | NNN | AAAATGGACTATCATATGCTTACCG |
| sgScreen_Fusion_F1.3 | ACACTCTTTCCCTACACGACGCTCTTCCGATCT | NNNN | AAAATGGACTATCATATGCTTACCG |
| sgScreen_Fusion_F1.4 | ACACTCTTTCCCTACACGACGCTCTTCCGATCT | NNNNN | AAAATGGACTATCATATGCTTACCG |
| sgScreen_Fusion_F1.5 | ACACTCTTTCCCTACACGACGCTCTTCCGATCT | NNNNNN | AAAATGGACTATCATATGCTTACCG |
| sgScreen_Fusion_F1.6 | ACACTCTTTCCCTACACGACGCTCTTCCGATCT | NNNNNNN | AAAATGGACTATCATATGCTTACCG |
| sgScreen_Fusion_F1.7 | ACACTCTTTCCCTACACGACGCTCTTCCGATCT | NNNNNNNN | AAAATGGACTATCATATGCTTACCG |
| sgScreen_Fusion_R1.1 | GTGACTGGAGTTCAGACGTGTGCTCTTCCGATC | NN | CCACTTTTTCAAGTTGATAACGGA |
| sgScreen_Fusion_R1.2 | GTGACTGGAGTTCAGACGTGTGCTCTTCCGATC | NNN | CCACTTTTTCAAGTTGATAACGGA |
| sgScreen_Fusion_R1.3 | GTGACTGGAGTTCAGACGTGTGCTCTTCCGATC | NNNN | CCACTTTTTCAAGTTGATAACGGA |
| sgScreen_Fusion_R1.4 | GTGACTGGAGTTCAGACGTGTGCTCTTCCGATC | NNNNN | CCACTTTTTCAAGTTGATAACGGA |
| sgScreen_Fusion_R1.5 | GTGACTGGAGTTCAGACGTGTGCTCTTCCGATC | NNNNNN | CCACTTTTTCAAGTTGATAACGGA |
| sgScreen_Fusion_R1.6 | GTGACTGGAGTTCAGACGTGTGCTCTTCCGATC | NNNNNNN | CCACTTTTTCAAGTTGATAACGGA |
| sgScreen_Fusion_R1.7 | GTGACTGGAGTTCAGACGTGTGCTCTTCCGATC | NNNNNNNN | CCACTTTTTCAAGTTGATAACGGA |

**Supplementary table legends**

**Table S3: sgRNA counts from the genome-wide screen. For** each sgRNA the ID, the ENTREZ ID, the gene symbol, its sequence and read counts for the baseline samples (GW_baseline 1 and 2), the non-phagocytosing (GW_low_1-3) andphagocytosing (GW_high_1-3) THP-1 cells and the plasmid (GW_plasmid) are given.

**Table S4: MAGECK-MLE analysis of the genome-wide screen.** Phagocytosing samples (high) were compared to non-phagocytosing samples. For each gene (SYMBOL) the ENTREZ ID the number of sgRNAs present in the screen and the statistical parameters are given comprising beta value, z score, raw p value, false discovery rate (fdr), p value using Wald test and the false discovery rate using Wald test.

**Table S5 sgRNA counts from the validation screen.** For each sgRNA the ID, the ENTREZ ID, the gene symbol, its sequence and read counts for the baseline samples (Val_baseline 1 and 2), the non-phagocytosing (Val_low_1-3) andphagocytosing (Val_high_1-3) THP-1 cells and the plasmid (Val_plasmid) are given.

**Table S6: MAGECK-MLE analysis of the validation screen.** Phagocytosing samples (high) were compared to non-phagocytosing samples. For each gene (SYMBOL) the ENTREZ ID the number of sgRNAs present in the screen and the statistical parameters are given comprising beta value, z score, raw p value, false discovery rate (fdr), p value using Wald test and the false discovery rate using Wald test.
